# Protocol: the complexity of informal caregiving for Alzheimer's disease and related dementias in rural South Africa

**DOI:** 10.12688/wellcomeopenres.18078.1

**Published:** 2022-08-25

**Authors:** Lenore Manderson, Michelle Brear, Farirai Rusere, Meagan Farrell, Francesc Xavier Gómez-Olivé, Lisa Berkman, Kathleen Kahn, Guy Harling

**Affiliations:** 1 School of Public Health, University of the Witwatersrand, Johannesburg, South Africa; 2 School of Social Sciences, Monash University, Clayton, Australia; 3 School of Public Health and Preventive Medicine, Monash University, Clayton, Australia; 4 MRC/Wits Rural Public Health & Health Transitions Research Unit (Agincourt), University of the Witwatersrand, Johannesburg, South Africa; 5 Harvard Center for Population and Development Studies, Harvard T.H. Chan School of Public Health, Boston, MA, USA; 6 Institute for Global Health, University College London, London, UK; 7 Africa Health Research Institute, KwaZulu-Natal, South Africa; 8 School of Nursing & Public Health, College of Health Sciences, University of KwaZulu Natal, Durban, South Africa

**Keywords:** Aging; Caregiving; South Africa; Dementia

## Abstract

Background: With aging, many people develop Alzheimer’s disease or related dementias (ADRD) as well as chronic physical health problems. The consequent care needs can be complicated, with heavy demands on families, households and communities, especially in resource-constrained settings with limited formal care services. However, research on ADRD caregiving is largely limited to primary caregivers and high-income countries. Our objectives are to analyse in a rural setting in South Africa: (1) how extended households provide care to people with ADRD; and (2) how the health and wellbeing of all caregivers are affected by care roles.

Methods: The study will take place at the Agincourt health and socio-demographic surveillance system site of the MRC/Wits Rural Public Health and Health Transitions Research Unit in Mpumalanga Province, northeast South Africa. We will recruit 100 index individuals predicted to currently have ADRD or cognitive impairment using data from a recent dementia survey. Quantitative surveys will be conducted with each index person’s nominated primary caregiver, all other household members aged over 12, and caregiving non-resident kin and non-kin to determine how care and health are patterned across household networks. Qualitative data will be generated through participant observation and in-depth interviews with caregivers, select community health workers and key informants. Combining epidemiological, demographic and anthropological methods, we will build a rich picture of households of people with ADRD, focused on caregiving demands and capacity, and of caregiving’s effects on health.

Discussion: Our goal is to identify ways to mitigate the negative impacts of long-term informal caregiving for ADRD when formal supports are largely absent. We expect our findings to inform the development of locally relevant and community-oriented interventions to improve the health of caregivers and recipients, with implications for other resource-constrained settings in both higher- and lower-income countries.

## Introduction

With increasing longevity, the prevalence of Alzheimer’s disease and related dementias (ADRD) is rising and will continue to do so rapidly ( Gallagher-Thompson *et al.*, 2012; Kalaria *et al.*, 2008; Nichols *et al.*, 2022; Prince *et al.*, 2013; Sosa Ortiz *et al.*, 2012). Rising dementia prevalence increases the need for informal caregiving, placing a heavy burden of physically, emotionally, socially and financially demanding work on family and friends ( Chiao *et al.*, 2015; Colvin & Bullock, 2016; Etters *et al.*, 2008; Merrilees, 2016). While caregiving may enhance family functioning and strengthen ties ( Liu & Huang, 2018; Pankong *et al.*, 2018), caregivers of people living with dementia report greater subjective burden and lower quality of life than other caregivers ( Karg *et al.*, 2018). Burden increases as dementia care recipients become more confused, emotionally labile, and unpredictable in behaviours and capability ( Dodel *et al.*, 2015; Rattinger *et al.*, 2015).

The health impact of dementia caregiving is well described in higher-income countries ( Conrad *et al.*, 2018; Park *et al.*, 2015; Williams *et al.*, 2008). However, by 2050 70% of those living with dementia and needing care will live in LMICs ( Prince *et al.*, 2013). In sub-Saharan Africa (SSA) prevalence of dementia is expected to almost double from 2019 to 2050, driven by longevity and population growth ( Nichols *et al.*, 2022). We suspect that existing findings will not translate in such settings, hence the need for context-relevant evidence.

Dementia caregiving is likely to have greater, more variable health impacts in sub-Saharan African and other LMICs than in higher-income settings. Financial or healthcare support for people living with dementia in LMICs is often absent or limited ( Button *et al.*, 2018), impacting the physical and mental health of caregivers and recipients ( Ramos *et al.*, 1993; Russell, 2009). Evidence regarding informal dementia caregiving in sub-Saharan Africa has been generated in several countries, including Kenya, Nigeria, Republic of Congo, South Africa, Tanzania, and Uganda. Such caregiving is associated with significant economic and social burdens. These burdens include increased financial costs ( Ainamani *et al.*, 2020; Mayston *et al.*, 2017) and support needs ( Smith *et al.*, 2020), as well as negative impacts on mental health ( Ainamani *et al.*, 2020; Gurayah, 2015), experiences of stigma ( Ainamani *et al.*, 2020; Nwakasi *et al.*, 2021; Musyimi *et al.*, 2021), quality of life ( Hendricks-Lalla & Pretorius, 2020) and family functioning ( Gurayah, 2015; Mayston *et al.*, 2017; Smith *et al.*, 2022). However, many facets of dementia caregiving in sub-Saharan Africa remain poorly understood. Notably, most research is focused on the primary caregiver – ADRD care recipient dyad, leaving very limited knowledge about the negotiation and division of caregiving responsibilities within and beyond households ( Smith *et al.*, 2022).

Households in LMICs and minority populations in higher-income countries are often multigenerational, frequently headed by older women ( Acheampong, 2016; Makiwane *et al.*, 2017; Muthwa, 1994; Simoes & Alberto, 2015), with strong social ties to kin and non-kin beyond households ( Burholt & Dobbs, 2014; Muthwa, 1994). Some LMIC households draw on these extended networks, but such networks may not suffice to moderate the impacts of dementia caregiving on the caregiver’s health nor to meet the need for formal support. Examining how dementia caregiving in LMICs is distributed within and beyond households, affecting the health of caregivers and others, will improve our understanding of informal care and its policy implications ( Burman, 1996; Makiwane *et al.*, 2017; Muthwa, 1994).

Rural South Africa provides a unique opportunity to examine the distribution and health impact of informal caregiving for people living with dementia in LMICs for several reasons. First, the dementia burden in rural South Africa is rising rapidly with population aging as in many middle-income countries, a pattern likely to follow elsewhere in SSA ( Nichols *et al.*, 2022). Second, both women and men in midlife in rural South Africa may migrate for work ( Camlin *et al.*, 2014; Collinson, 2010), leaving smaller households with fewer adults to provide substantive care to others ( Collinson *et al.*, 2007). Common chronic health problems including HIV and cardiometabolic disease may likely limit what care many of these adults can provide ( Gaziano *et al.*, 2017). Care therefore may be shared among prime-aged and older adults, grand- and great-grandchildren. Third, resources supporting informal caregivers in this setting are limited, with no direct government financial support ( Ralston *et al.*, 2016). Government programs for chronic care for HIV, hypertension and diabetes have grown ( Ameh *et al.*, 2017a; Ameh *et al.*, 2017b), but formal home- and facility-based care for dementia is largely absent ( Goudge *et al.*, 2009; van der Hoeven *et al.*, 2012). Fourth, potential sources of resilience for informal caregivers vary across the population. This includes both kin and non-kin social connections, offering financial, emotional, and instrumental support and cognitive reserves that may buffer emotional strain ( Harling *et al.*, 2020; Humphreys *et al.*, 2017; Kobayashi *et al.*, 2017; Winchester & King, 2018). However, support likely varies seasonally and with circular and out-migration.

## Protocol

The current protocol is version 1.0 dated 5 March 2020.

### Study aims

Our objective in this study is to document how people in rural South Africa negotiate and provide informal care to people with ADRD, and how they are affected by this caregiving. Our specific aims and research questions are:

1) To determine who is delivering what care to older adults living with ADRD.

a) How is caregiving for those living with ADRD shared across household social networks, among resident and non-resident kin and non-kin?
b) Is caregiving involvement moderated by loss/absence of working-age adults and access to formal care?
c) How do caregiving activities vary according to the care recipient’s severity of dementia, behavioural changes, and physical health?

2) To establish the impact of AD and other dementia care on the social, mental, and physical wellbeing of caregivers and people connected to them.

a) How do the health impacts of ADRD differ for resident caregivers, resident non-caregivers, and non-resident caregivers?
b) How does access to economic and healthcare resources mitigate the health impacts of caregiving, and how do households strategize to access these resources?
c) How does availability of support through social networks protect caregiver’s and resident non-caregivers’ health, and how does caregiving affect social networks?

### Study design

The study, referred to as Kaya (the word for home or residence in Shangaan/Xitsonga, the primary language in the study site), will be a mixed methods design. Quantitatively, we will conduct cross-sectional survey interviews with actual and potential caregivers, compromising the social networks of ~100 rural South African households with a resident with ADRD. Qualitatively, we will conduct ethnographic work in a subsample of households and undertake key informant interviews.

### Setting

Kaya is nested within the Agincourt Health and Socio-Demographic Surveillance System (HDSS) site of the Medical Research Council/University of the Witwatersrand Rural Public Health and Health Transitions Research Unit, in Bushbuckridge, Mpumalanga province, South Africa. The Agincourt HDSS has maintained an annual census of ~120,000 people in 31 rural Mpumalanga communities since 1992 ( Kahn *et al.*, 2012). Households have high but variable stress levels; 17% have lost family members to AIDS ( Schatz *et al.*, 2011; Schatz *et al.*, 2015). Adult circular migration is common, with working adults returning periodically for holidays or because they are sick and need care ( Clark *et al.*, 2007; Collinson, 2010; Collinson *et al.*, 2007).

The Agincourt HDSS provides a sampling frame for numerous epidemiological studies, including HAALSI (Health and Aging in Africa: A Longitudinal Study of an INDEPTH Community in South Africa), which commenced in 2014 interviewing a random sample of 5059 adults aged ≥40 ( Gómez-Olivé *et al.*, 2018). HAALSI completed its third data collection round in 2022.

The HAALSI Dementia Study began in 2019–20 to estimate prevalence and incidence of dementia and mild cognitive impairment (MCI) in the HAALSI cohort. Its baseline survey recruited 635 HAALSI respondents based on a stratified sample that oversampled those at higher risk for dementia based on brief cognitive screening scores obtained in the second wave of the parent HAALSI study. HAALSI Dementia respondents completed rigorous cognitive testing, informant interviews, and a clinical neurological examination to evaluate neurocognitive deficits and functional limitations. An expert clinical panel reviewed summarized findings from cognitive tests, informant interviews, and neurological exams and assigned consensus diagnoses of normal cognitive function, MCI, or dementia based on National Institute on Aging-Alzheimer’s Association (NIA-AA) 2011 criteria ( McKhann *et al.*, 2011). Full details on the protocol have been published ( Bassil *et al.*, 2022). The expert panel additionally provided dementia severity ratings based on the Clinical Dementia Rating scale (0–3 ( Morris, 1997). A second Dementia Study wave was conducted in 2022 adding a refresher sample to account for death and attrition.

### Participant selection and sampling strategy 

We will sample as index cases 100 HAALSI Dementia Study Wave 2 participants known or predicted to have dementia. We will include all wave 1 participants who were diagnosed with moderate or severe dementia at baseline and remain in the cohort with dementia. Since clinical diagnoses for Wave 2 HAALSI Dementia Study participants are not available for Kaya sampling, we will identify additional participants by predicting each Wave 2 respondent’s probable clinical diagnosis severity score using the relationships between cognitive/informant measures and clinical consensus diagnoses observed in Wave 1. First, we will construct a multinomial logit model using selected variables from Wave 1 to predict a 3-level dementia severity score based on consensus diagnoses at wave 1: 1 (no dementia), 2 (mild dementia), or 3 (moderate to severe dementia). This model correctly predicted Wave 1 clinical dementia diagnosis severity category with 92% accuracy. We will then apply coefficients from the prediction model to the cognitive/informant measures collected in Wave 2 to obtain each respondents’ predicted clinical severity category at Wave 2. This measure is intended to capture which category the expert panel is likely to assign them to, based on currently available information. Our process is modelled on past work using similar methods to algorithmically ascertain dementia status when clinical ratings are not available ( Crimmins *et al.*, 2011; Gianattasio *et al.*, 2020). We will stratify factorially by predicted level of dementia (Clinical Dementia Rating 1 [mild] vs 2–3 [moderate or severe]) and gender, since care receipt is likely to differ substantially on these axes; within each cell we will sample 25 index cases.

For each index case, we will conduct quantitative interviews with household members aged ≥12 and non-resident caregivers (~1000 individuals across 100 households). We are including minors aged 12–17 since they are likely to play an important role in caregiving and having a household member living with ADRD is likely to have a substantial impact on their wellbeing. Children under 12 will be excluded from participation in the quantitative study, but are likely to be present in the 20 households within which participant observation will be conducted. While there are no gender restrictions on participants, we expect more women than men to participate, based on the gender balance of research site residents and the gendered nature of caregiving. Should fewer than 1000 individuals be identified by enumerating the networks of 100 households we will sample additional cases until 1000 interviews have been completed.

For the qualitative study, we will purposively subsample 20 households (5 from each cell) for maximum variability sampling in: care recipient and provider demographics; economic situation (current status and past shocks); care experience; and household and social network composition (household size, stability of household membership, generational structure) ( Etkind *et al.*, 2019; Gozzoli *et al.*, 2018; Surakarn *et al.*, 2016). This diversity will maximize confidence and generalizability ( Sykes *et al.*, 2018). We will conduct repeated semi-structured in-depth interviews and participant observation in these households.

These data will be supplemented by ~30 key informant interviews with church and community leaders and with staff in: provincial and district level departments of Social Development and Health; NGOs and community organizations concerned with ageing and chronic health conditions; and day care and residential care centres. Additional interviewees will be identified through discussion with the Agincourt Public Engagement Team and interviewees (respondent-driven sampling) ( Heckathorn & Cameron, 2017; Mmari *et al.*, 2016; Nesterko & Blitzstein, 2015).

## Measurements

### Quantitative data

**Figure 1.**
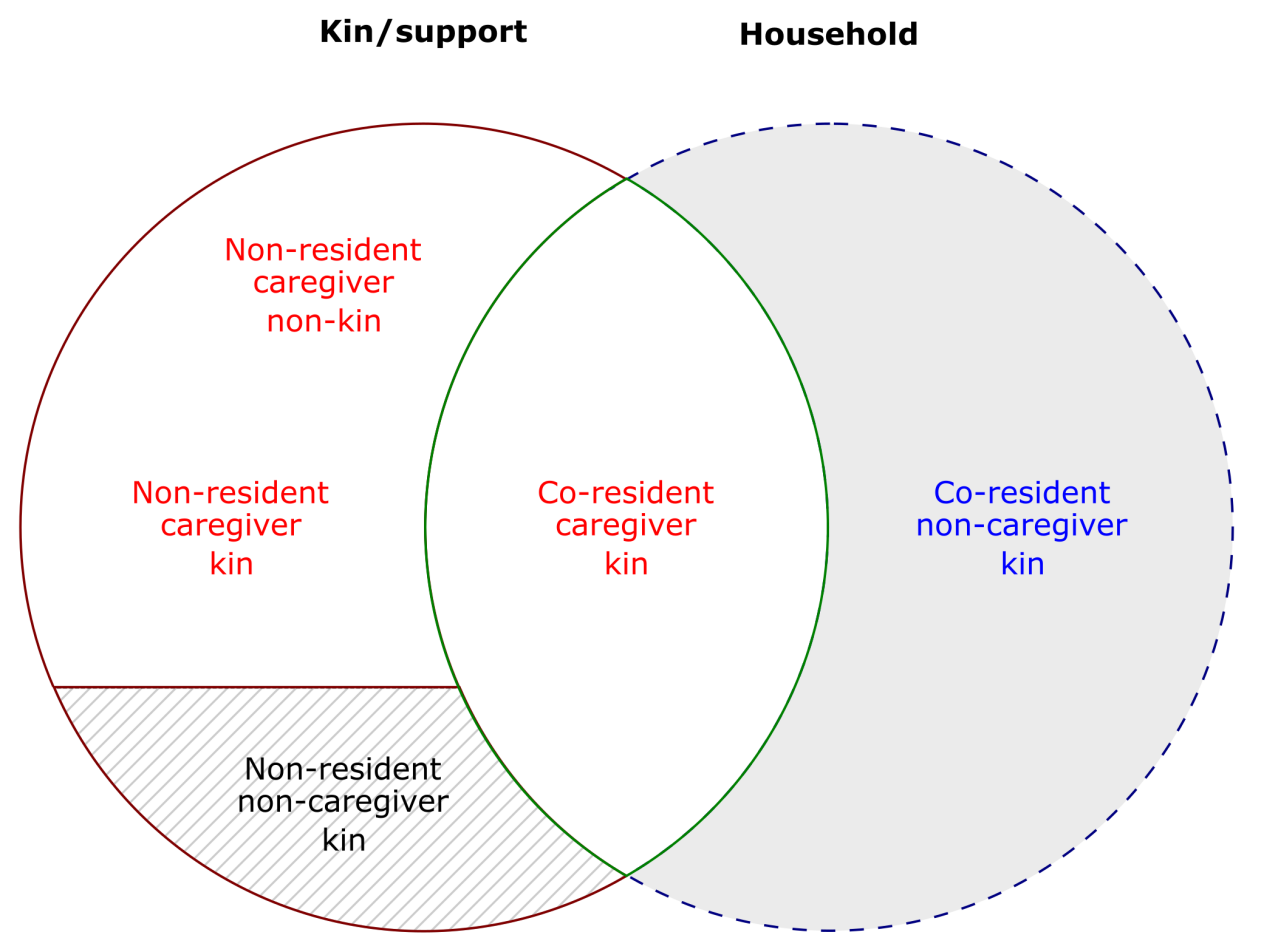
A schema of study participants. We will interview all caregivers (in white areas) and co-resident non-caregiver kin (grey area). Adapted from Madhavan *et al*., 2017.

The identified primary caregiver for each index case will be invited to participate in a survey as the “initial respondent” for the household. This will involve enumerating household members, and non-resident kin and kith who actually or may potentially participate in dementia caregiving ( Figure 1) ( Madhavan *et al.*, 2017). The initial respondents will then be asked to provide sufficient identifying information about named individuals that they can be contacted and invited to participate in the study. The initial respondent will also be asked to evaluate the health and wellbeing of the index person living with ADRD.

The initial respondent, and all eligible individuals named by the initial respondent (same household or caregivers) will be asked to complete a longer questionnaire, covering:

- Individual socio-demographics, including economic situation;
- Personal social network. This module will focus on capturing important social relationships, including information on provision/receipt of emotional, informational, physical, financial and socialization support, and details on the nature of the relationships. It will also capture identifiable information on all persons identified, so that the social network of the household can be mapped across respondents;
- Caregiving, including care provision time, perceived burden and the disruptiveness of caregiving;
- Caregiving-specific wellbeing, including mental, social and financial impacts of caregiving and coping strategies used; and
- General health and wellbeing, including physical and mental wellbeing and healthcare need and utilization.

### Qualitative data

In-depth interviews will examine task sharing, time spent on care, caregiver’s health and wellbeing, household and family relationships, decisions about and fluidity of care, impact of extra-household events (work opportunities, other illness, in- or out- migration), responsibility and relationality, and interpersonal pressures with attention to variation, complexity and change. We will examine how care work varies by gender, age, kinship, and economic participation; positive and negative changes in interpersonal relations in and beyond households; informal support through networks; household and structural impediments to formal care. We will also explore variations in care needs and provision within and between households, household size, composition, relatedness, and age of caregivers.

In key informant interviews we will explore: provision of key of people with dementia in community settings; frequency of house visits; support to caregivers; provision of community care through churches and other agencies; role and function of respite/residential care (one NGO provides residential care in the study setting).

## Data collection and management

### Consent procedures

All informed consent/assent forms will be written in English, translated into Shangaan/Xitsonga, then back translated and revised for accuracy, clarity and ease of comprehension. Standard study information – including aims, risks, benefits, confidentiality, time, and nature (including voluntariness) of participation and the ongoing right to withdraw – will be provided in writing, read to or discussed with each potential participant. The discussion will be conducted in Shangaan or English. For those who choose to participate, informed consent and assent will be documented electronically. Participants will be provided with a paper copy of the information sheet and informed consent document in their preferred language. Any eligible minors (12–17 year olds) will only be approached for assent to participate once a parent or guardian has provided informed consent for their child/ward to participate. In line with standard Agincourt HDSS policies, no financial payment will be made for participation in this study.

### Quantitative data

Quantitative data will be entered and stored in the field on encrypted electronic tablet computers using REDCap ( Harris *et al.*, 2019) and Network Canvas ( Birkett *et al.*, 2021) software. On return to the Agincourt HDSS field office, the data will transferred to a secure server using end-to-end encryption. The data will be regularly checked for outliers and completeness, and automated data checks and limits are built into the data capture software. Prior to releasing analytic datasets, the Data Management team at Agincourt will remove all identifier variables, replacing them with their unique identifier in the Agincourt HDSS system, which will allow linkage to other HDSS data using a key held only by the Head of Data Section at Agincourt. These pseudonymized data will then be exported from the secure server to study team members for analysis.

### Qualitative data

For qualitative work, household members will usually be interviewed in Shangaan/Xitsonga, while key informants will be interviewed either in Shangaan or English. Audio recordings of formal interviews will be made using digital recorders and transcribed and translated. Observations and informal conversations will be carefully noted directly into Word documents on study team members’ laptops. All interviews and field observation notes will be stored as separate Word files, with descriptions of content and codes to link this qualitative data to the survey data. Digital recorders and laptops will be encrypted at rest and kept in a secure location within the Agincourt office.

Back-up procedures will ensure that data interview files and source documents are securely stored; electronic data will be stored in two or more locations, including a local server and on an encrypted secure website dedicated to the project data. Once securely backed up to the latter locations, the data will be cleared from the original device.

All Word documents will be imported to NVivo 12 software ( QSR, 2019) to organize, store and retrieve data, including typed text, transcribed audio records, video, survey data, and primary and secondary source material. Preliminary coding and analysis will begin from the first interviews to enable iteration, and transcripts will be reviewed against audio-recorders to check for quality and completeness. Analysis will follow established qualitative techniques such as thematic and interpretive analysis.

### Data storage and dissemination

Both quantitative and qualitative data will be integrated into Agincourt’s existing data management systems. These include a structured chain-of-evidence for all paper-based records, and the use of locked rooms, secure computers and strict monitoring of access to rooms containing records or computers with personal data. Only senior research team members and necessary field staff will have access to datasets containing both participant pseudonymization keys and personally identifiable data on participants, and only on a need-to-know basis. We envisage this being needed only for data collection operational procedures and for data analysis across linked datasets. Only aggregate data will be reported in dissemination, with pseudonyms for individuals and for place names.

Data files will be stored on a password-protected server in a room with controlled physical access at Agincourt for the duration of the project, with regular back-up copies made on password-protected file space. All data will be stored for up to 20 years from study close, with the pseudonymization key maintained on the password-protected university computer system. Long-term data storage will be in .csv files using a standard (simple) ASCII coding scheme for survey data, and in .rtf files for qualitative data.

The survey data and transcripts from formal in-depth interviews will be available 12 months after locking the analytical database for access by bona fide researchers. Consent for such sharing will be obtained from participants at initial informed consent. Participant observation data notes and informal interviews will not be shared, as consent cannot anticipate all interactions and incidents in the context of observation. Pseudonymized data will be made available for download following a signed data use agreement (stating the purpose of use and agreeing to maintain data protection) from the MRC/Wits-Agincourt Unit data repository ( http://www.agincourt.co.za/index.php/data/). Anonymized data will additionally be placed in the DataFirst archive and made available to researchers through the data portal ( https://www.datafirst.uct.ac.za/). Prior to such data releases, special statistical identity disclosure protection measures will be used including, but not restricted to, data anonymization, recoding and aggregation.

### Data linkage

Data collected in Kaya will be linked to previous data collected during the Agincourt HDSS census, HAALSI survey rounds and the HAALSI Dementia study. Linkage will also be made to local public sector clinic care records, via an existing linkage system. All these linkages will be made using each participants unique Agincourt HDSS identifier or study-specific unique identifiers. This linkage will be conducted by the Agincourt data section team in an access-controlled environment office. The linked data will be managed in the same manner as newly collected data from this study. Asset and consent for this data linkage will be requested from all study participants during the initial informed consent process.

## Data analysis

We will use a combination of qualitative, quantitative, and mixed method techniques to analyse the data. Mixed method analysis integrates results or data collected using different methods, to produce “meta-inferences” that constitute more than the sum of the (quantitative plus qualitative) parts ( Fetters & Freshwater, 2015). It is valuable for studying complex research questions that cannot be adequately answered using one method alone ( Morse, 2016). Mixed method analysis approaches that we will use include:

- *Cross-over mixed analysis*, in which qualitative data are analysed quantitatively or vice versa. (e.g. quantify qualitative social network data, and comparing to quantitative findings) ( Hitchcock & Onwuegbuzi, 2022);
- *Qualitatively driven mixed-method analysis,* in which quantitative results are used to supplement qualitative findings ( Morse, 2016); and
- *Triangulation,* in which quantitative and qualitative data and findings are considered in relation to each other, to enhance the depth and rigor of conclusions drawn, as described below ( Denzin, 2012).

Quantitative personal social network data relating to a single index person will be connected based on the identifying information provided to generate a household-level “sociocentric” social network. For all aims, our quantitative analysis will be based on two-level multilevel models (respondents nested within households). From this network several measures typifying the household will be calculated, including the size of network, the density of connections and support provision and the proportion of connections that are within the household and between kin. We will use network-specific imputation and modelling methods to analyse the impact of such missing data if missingness is substantial ( Huisman & Steglich, 2008; Wang *et al.*, 2016). For both quantitative and qualitative analyses, we will account for the gender, age and HIV serostatus of respondent and person living with ADRD, as well as household size and composition,

For aim 1a, we will quantitatively test whether residency and/or kin status predict caregiving burden. Before conducting statistical regression analysis, we will describe the proportion and kinds of care provided by residents/non-residents and kin/non-kin. Qualitatively, we will illustrate how care is negotiated and concentrated with non-kin and non-resident support.

For aim 1b we will qualitatively link changing kinship relations and family composition to fluctuations and adaptations in caregiving patterns. This analysis will be supported with tests for effect-modification of the association of residency/kin status and caregiving burden by household-level variables (e.g., size and composition) using cross-level interaction terms in multilevel models.

For aim 1c we will qualitatively identify how changes in care recipients’ function and capability influence demand for, and desire and capacity to provide, care – expecting seasonal fluctuations in care to affect all these. Quantitatively, we will test for effect-modification of associations between residency/kin status and care by recipients’ cognitive status.

For aim 2a we will quantitatively test the hypothesis that caregivers have more health concerns than non-caregivers and determine whether this difference is most pronounced for those with the greatest caregiving burden (i.e., objective and subjective caregiving burden will effect-modify the difference in health between caregivers and non-caregivers). For all aim 2 quantitative outcomes, we will analyse each measure of health separately, accounting for multiple hypothesis testing and using Seemingly Unrelated Regression to account for interdependencies across wellbeing types ( Zellner, 1962). Interviewees narratives of decision-making to care, negotiations among kin and non-kin, and embodied experiences associated with care work will add depth to the quantitative data.

For aim 2b we will map the effects on households of limited resources and the particular burden on those providing most care using qualitative data, illustrating the consequences of and adaption to demographic and economic changes. Quantitatively, we will test for effect-modification of associations between level of caregiving burden and wellbeing by household wealth (asset index from HDSS), migrancy count, recent mortality (past 24 months), ability to access formal healthcare if needed and healthcare use in past 12 months. We will run separate regression models for each potential stressor/effect-modifier (wealth, migration, mortality, healthcare use) measured at index-household level, testing cross-level interactions of individual caregiving burden and each household stressor. We expect limited resources to have most effect on the wellbeing of those providing most care.

For aim 2c we will quantitatively test the hypothesis that respondents’ wellbeing is greater when household-level social networks are larger, denser and more diverse (e.g., by age, location, support shared). We will test whether this effect is mediated by more received emotional, financial or physical support and lower self-reported unmet need for such support, using the Natural Indirect Effect ( Lange *et al.*, 2012). We will also assess how associations between stronger social networks and caregiver health are effect-modified by access to beyond-household-network informal or formal care and caregiver impairment status for basic and instrumental activities of daily living. Qualitatively, we will explore how support is secured through varied kinship and friendship networks; how support systems assist caregivers to meet their own and health needs and those of the person living with dementia; and how social networks and care support atrophy or expand as care needs change over time with fluctuations in the status of the person living with dementia and in relation to others’ needs.

## Sample size and statistical power

**Figure 2.**
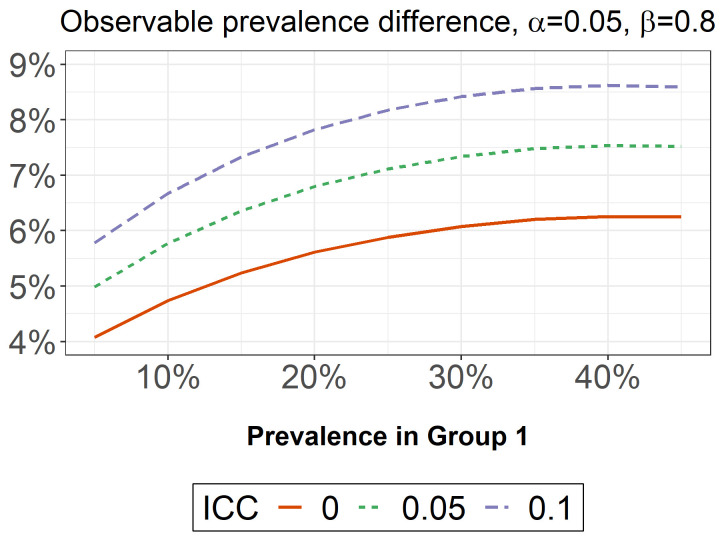
With an intraclass correlation coefficient of 10%, our sample provides 80% power to detect prevalence differences of <9% between two equally sized groups.

Our power calculation aimed to ensure with 95% confidence that our quantitative sample would be sufficient to provide 80% power to see a 10-percentage point difference in the proportion of two equal-sized groups on any binary outcome when the outcome prevalence was around 50%. We calculated this based on a conservative intraclass correlation coefficient (ICC) of 10% (design effect of 1.9) given a sample of 1000 individuals across 100 households. Our key outcomes of interest include caregiving load (Aim 1) and caregiver wellbeing (Aim 2), Should the outcome be rarer/more common, or the ICC smaller (e.g., if we have to extend beyond 100 households), then the minimum detectable difference will be lower ( Figure 2) ( Cohen, 1988). If we treat caregiving load or wellbeing as a continuous variable, power will be higher.

Power for interactions cannot easily be calculated a priori, since it relies on all variables’ intercorrelations and we do not have preliminary data on this. We will therefore focus primarily on qualitative findings for aims involving effect-modification, supported by quantitative data as available. Data from this study will inform power calculations for future longitudinal work.

For qualitative data collection, sample sizes were chosen based on past experience of the quantity of data required to reach saturation. Precise decisions on when to stop data collection will be based on team discussions conducted as fieldwork progresses.

## Ethical consideration

Ethics approval for this research has been provided by the University of the Witwatersrand’s Human Research Ethics Committee (M200373), University College London Research Ethics Committee (15231/011) and Mpumalanga Province Health Research Ethics Committee.

## Participant protection

The study population will be representative of the Agincourt community, and may therefore include vulnerable populations who are elderly, legal minors aged 12–17, economically disadvantaged, illiterate, cognitively impaired or living with HIV. The rights and welfare of all participants will be safeguarded through informed consent and privacy procedures. Since some of the topics that will be covered are sensitive, several steps will be taken to minimize the risk of loss of confidentiality. All interviews will be conducted in locations from where discussions cannot be overheard by others; data will be collected on electronic devices that are encrypted at rest; and data will be pseudonymized prior to storage on a secure server. Staff will be trained to minimize the risk of psychological distress during the interview, and will follow a standard operating procedure for seeking assistance if any participants become distressed as a result of participation in the research.

## Community engagement

Community engagement will occur before, during, and after the research project, through existing structures. The Agincourt unit has been working with a Community Advisory Board CAB since 2007, whose members are nominated by an elected village leadership structure, from each of the villages in the study area. All proposed study instruments will be discussed with the CAB and piloted prior to final use. The CAB develops appropriate research result dissemination materials and strategies to ensure the community is aware of the purpose of the work. Engagement activities for Agincourt HDSS already include: annual community feedback meetings at village and service-provider levels; community newsletters; development of simple, translated ‘fact sheets’ containing population data and study findings (distributed at community meetings, and discussed with village leaders, fieldworkers and service-providers); a website; facilitation of the CAB; and addressing community concerns.

We will also engage with the Mpumalanga Department of Health, local and provincial NGOs concerned with aging and caregiving, and the national Ministry of Health. In past meetings, provincial and district government departments have expressed direct interest in elderly care, particularly for people living with ADRD.

## Social network identification

In naming and providing information about social contacts, survey respondents will provide information about people who have not consented to participate. Such information might be private or cause social harm if passed to others in the community. However, revelation of third-party identities and behaviours is common (e.g., when conducting proxy interviews with household heads, or asking about sexual behaviour with spouses), and it has been argued such information is respondents’ to give, i.e., respondents are providing their view of relationships with others ( Klovdahl, 2005). From an ethical standpoint, provision of third-party information may be considered acceptable when: (i) no other method is available; (ii) third party identities are maximally protected; and (iii) benefits outweigh harms ( Lounsbury *et al.*, 2007). We believe that these conditions are met in this study since: (i) as for any sociocentric analysis, only by asking respondents to identify their contacts can a social map be built; (ii) a range of identity protection methods are in place as outlined above; (iii) the social dynamics captured through social network analysis are central to understanding how caregiving is provided and how it impacts households and beyond, providing a strong potential of benefit to many community members.

## Staff safety

To optimise field staff safety, transport to participant homes will be in Agincourt HDSS vehicles which are serviced regularly. Fieldworkers’ locations will be logged daily in a central off-site register and in real-time in a WhatsApp group overseen by the field coordinator, who will conduct regular debriefings of field teams to discuss past and potential future concerns. Senior researchers will conduct regular debriefings with members of the research team.

## Discussion

The work in this mixed methods study will provide important and novel information on how caregiving is shared across the extended social network of households affected by ADRD in a rural resource-limited setting. It will also provide information on how caregiving differentially impacts on the health of the extended social network of households affected by dementia in a high-poverty social setting.

Our findings will lay the basis for future longitudinal research on the dynamic changes in household caregiving and how they affect caregiver health. The work will support the development of interpersonal, community and structural interventions to support home-based care providers and improve the health of both caregivers and care recipients. Such interventions might include government support for in-home, day and residential care by health workers. Our work will identify key kinds and times of support and identify the most vulnerable individuals and households to whom such support should be directed, something that could potentially lead to self-screening tools for the community. Finally, we may be able to identify untapped potential sources of caregiving within social networks, for whom health messaging might be developed.

## Study status

The study commenced data collection on July 21 ^st^, 2022 and is expected to continue through the rest of the year.
