# Supplementary material for: Protocol: the complexity of informal caregiving for Alzheimer's disease and related dementias in rural South Africa
Source: Wellcome Open Res. 2022 Aug 25;7:220. [Version 1] doi: 10.12688/wellcomeopenres.18078.1 (PMC10394391; doi:10.12688/wellcomeopenres.18078.1)
Supplement: Peer review history

# Peer review: Reviewer response for version 1

**DOI:** 10.21956/wellcomeopenres.20048.r60683

**Published:** 2023-08-01

**Authors:** Martina Roes

**Affiliations:** 1 German Center for Neurodegenerative Diseases eV, Witten, Germany

**Abstract**:

- "With aging, many people develop Alzheimer’s disease or related dementias (ADRD)…" - other related dementias are not per se age related, thus a misleading start of the 1 ^st^ sentence in this paragraph.
- "We will recruit 100 index individuals predicted to currently have ADRD or cognitive impairment" - Has this been done by a medical doctor or how do you know?
- Objective - "in a rural setting in South Africa"; 
  Discussion - "with implications for other resource-constrained settings in both higher- and lower-income countries"; 
  Introduction - "We suspect that existing findings will not translate in such settings, hence the need for context-relevant evidence"
  Based on these pre-assumptions I’m not sure if will be possible to provide details for "other recourse-constraint settings in both higher- and lower income countries’ (abstract discussion)"?

**Introduction**

- "Burden increases as dementia care recipients become more confused, emotionally labile, and unpredictable in behaviours and capability" - The pic of plwd presented here is a negative one and does not acknowledge that having any form of dementia does not per se, lead to the mentioned ‘negative behavioral symptoms’. People with lewy body for example do not experience (for a long time) changes in their emotional expression or cognitive capacities. Not all types of dementia are emotional labile (there might be a link to depression too). Please be careful about generalization of symptoms linked to dementia.
- "...leaving smaller households with fewer adults to provide substantive care to others ( Collinson et al., 2007)" - Aren’t there newer data/publications?
- "...but formal home- and facility-based care for dementia is largely absent ( Goudge et al., 2009; van der Hoeven et al., 2012)" - Aren’t there newer data/publications?

**Protocol**

- "The current protocol is version 1.0 dated 5 March 2020" - Not sure what this means?

**Study design**

- "Shangaan/Xitsonga, the primary language in the study site" - I did not see this mentioned in the Study Aims, you may add the rural area or name of the study site. For example: To determine who is delivering what care to older adults living with ADRD in Bushbuckridge, Mpumalanga province, South Africa.
- "cross-sectional survey interviews" (section: Study Design) versus "will be invited to participate in a survey" (section: Quantitative Data) versus "conduct quantitative interviews" (section: Participant selection and sampling strategy) - Please be consistent in your wording.
- "Qualitatively, we will conduct ethnographic work" - I’m missing here the in-depth interviews and observations mentioned later on.

**Settings**

- From my understanding the 2 ^nd^ and 3 ^rd^ paragraph do not fit here. It’s not really about the setting / not about a description of the rural area, but rather details about data collected elsewhere (Wave 1?)

**Participant selection and sampling strategy**

- 1 ^st^ paragraph: Description of wave 1 and 2 is confusing, please clarify. Maybe provide a table instead of written paragraph?
- I’m missing recruitment strategies (i.e., how to reach out, timeframe etc.) after sampling of 100 HAALSI index cases have been identified. How do you identify church and community leaders. Who represents the church? Who the community? (possible selection bias / impacts data interpretation?)

**Measurements**

- I think this is more about data collection. By measurements I expect outcome measures, which I’m not seeing here.
- *Quantitative data:* The survey will be done in person? Or virtually? Who is the person who collects the data (students? Junior/senior researchers/research assistants/others?). Or will the survey be sent by mail and needs to be returned by mail?
- *Qualitative interviews with informal caregivers / with key informant:* Who is the person who collects the data (students? Junior/senior researchers/research assistants/others?) will the interview be conducted in their home or virtually or by phone?

**Consent procedures**

- Who will be involved in the forward-backward translation of the material (are all involved researchers bilingual)? How do you avoid biases if you first start with the English version (English as the reference language?) Why don’t you start with the native language, if interviews will be conducted in Shangaan/Xitsonga? Please provide a few more details.

*Qualitative data:*

- What do you mean by ‘formal interviews’ interviews?
- "Observations and informal conversations will be carefully noted directly into Word documents on study team members’ laptops." - Does this mean that there are 2 researchers during the interviews? (1 = interviews and 1 = observations?)
- Will you use specific criteria for the observation?
- "Preliminary coding and analysis will begin from the first interviews to enable iteration" - I’ve not seen this in the study design section

**Data storage**

- "survey data and transcripts from formal in-depth interviews will be available 12 months after" - What about the observational and the key-informant data?

**Data analysis**

- Description is heavy on the quantitative analysis, missing more details about the qualitative data (ethnographic study and observational data, in-depth interviews, interviews with key informant).
- What about details about the mixed-method analysis?

**Ethical consideration**

- Is this from the overall wave 1 and 2 study?

**Community engagement**

- To better understand how embedded the study is within the community this section could be mentioned earlier.

**Social network identification**

- This might be added to the ethical consideration section.

**Discussion**

- "in a rural resource-limited setting" - add in Bushbuckridge, Mpumalanga province, South Africa.
- "in a high-poverty social setting..." - I’ve not seen you providing details on the socio-economic situation of the rural area earlier in the manuscript.

**Study status**

- Seems like data collection has been finished in 2022?

Is the study design appropriate for the research question?

Yes

Is the rationale for, and objectives of, the study clearly described?

Yes

Are sufficient details of the methods provided to allow replication by others?

Partly

Are the datasets clearly presented in a useable and accessible format?

Partly

Reviewer Expertise:

Dementia care research

I confirm that I have read this submission and believe that I have an appropriate level of expertise to confirm that it is of an acceptable scientific standard, however I have significant reservations, as outlined above.

# Peer review: Reviewer response for version 1

**DOI:** 10.21956/wellcomeopenres.20048.r60704

**Published:** 2023-07-04

**Authors:** Jennifer Bray

**Affiliations:** 1 University of Worcester, Worcester, England, UK

I think that this study will provide useful and important information, but I have a few queries and suggestions that could be addressed.

 I answered 'partly' for the sufficient details question as I feel there is a need for more clarity around selection process and measures used, as mentioned in the comments below.

 I did not see any datasets, or see how there could be any when the study has not been carried out. If I've missed something or misunderstood what was meant, I apologise.

 Introduction

- I would be careful about some of the language you use. I appreciate that you acknowledge a couple of positives from caregiving, but the use of ‘heavy burden’ and ‘burden’ gives an automatically negative view of caregiving. I’m not saying that you should remove all uses of ‘burden’, especially if it is how it is reported in the literature you are referring to, but maybe consider whether you could rephrase some bits with a less emotionally charged word to avoid influencing the reader. Your study aims seem to be quite balanced and neutral, i.e. you’re not focusing on burden or negative impacts, so it would be good if you could be a bit more balanced when setting up the background to the study.

Participant selection

- I find the first paragraph in this section quite confusing. You say you’re sampling 100 people from Wave 2, which is fine. You then say you’re including all relevant people from Wave 1 – is this in addition to the 100 from Wave 2, or are these the same people? You then say you will identify additional participants from Wave 2 – again, is this in addition to the 100 people from Wave 2 and potentially the relevant people from Wave 1? I’m assuming that you’re actually describing the process that you’re going to undertake to get your sample of 100 people, but at the moment it (to me) doesn’t sound like this. Also, at the end you say ‘within each cell’ but you don’t say what the cells are. I think this paragraph would benefit from some reordering and clarification to make the selection process clearer.
- I think there could be more consistency and clarity when describing what you are actually doing. In the abstract you refer to quantitative surveys, but in the study design section this has become survey interviews and in the participant selection section it is quantitative interviews. When talking about the measurements it has gone back to being a survey for the initial respondent, then is referred to as a longer questionnaire for all participants. What are you actually doing and how is it being administered? Is it a survey that people are left to get on with, or is it a survey that you go through with people during an interview?

Measurements

- Are any existing measures used to capture the information about caregiving, caregiving-specific wellbeing and general health and wellbeing, or did you design your own questions?

Staff safety

- It’s not clear whether fieldworkers will be working alone or in pairs. If alone, do you have a specific lone worker policy or procedure beyond keeping a register of locations and a WhatsApp group? For example, making it clear that a person sends a message before going into someone’s home and another when they have finished, and a coordinator acknowledges each message? At present it just feels a bit vague about how staff are protected.

**Minor points**

- You use the abbreviation LMIC without saying what it means.
- Why did you bother setting up the SSA abbreviation if you don’t use it on at least three occasions in the subsequent paragraph?
- In the participant selection section you say ‘wave 1’ not ‘Wave 1’ on a couple of occasions.

Is the study design appropriate for the research question?

Yes

Is the rationale for, and objectives of, the study clearly described?

Yes

Are sufficient details of the methods provided to allow replication by others?

Partly

Are the datasets clearly presented in a useable and accessible format?

Not applicable

Reviewer Expertise:

Dementia

I confirm that I have read this submission and believe that I have an appropriate level of expertise to confirm that it is of an acceptable scientific standard, however I have significant reservations, as outlined above.

# Peer review: Reviewer response for version 1

**DOI:** 10.21956/wellcomeopenres.20048.r53013

**Published:** 2022-11-09

**Authors:** Adam R. Roth

**Affiliations:** 1 Department of Sociology, Oklahoma State University, Stillwater, OK, USA

This protocol is well written and clearly explains the purpose and procedures of the proposed study. I have only a few minor suggestions. 

 On pg. 4, it would be nice if the authors mention the number of proposed participant observations in the first paragraph of Study Design. In the last paragraph of that page, they say that "...likely to be present in the 20 households within which participant observations will be conducted." Having not seen the number 20 prior to this, I was a little confused at first. 

 Clarify what is meant by "5 from each cell" on the top of pg. 5.

 On pg. 5 under Quantitative Data, the authors use the term kith but in Fig 1 (and elsewhere) they use the term non-kin. I suggest changing these terms for sake of congruency.

 On pg. 7, the authors explain how they will create household sociocentric networks. What about any non-household network members who were named as part of the personal network module. Will they be omitted from these sociocentric networks?

Is the study design appropriate for the research question?

Yes

Is the rationale for, and objectives of, the study clearly described?

Yes

Are sufficient details of the methods provided to allow replication by others?

Yes

Are the datasets clearly presented in a useable and accessible format?

Yes

Reviewer Expertise:

Social network analysis, aging

I confirm that I have read this submission and believe that I have an appropriate level of expertise to confirm that it is of an acceptable scientific standard.
